# Fast and Efficient 5′P Degradome Library Preparation for Analysis of Co-Translational Decay in Arabidopsis

**DOI:** 10.3390/plants10030466

**Published:** 2021-03-01

**Authors:** Marie-Christine Carpentier, Cécile Bousquet-Antonelli, Rémy Merret

**Affiliations:** 1 CNRS-LGDP UMR 5096, 58 avenue Paul Alduy, 66860 Perpignan, France; marie-christine.carpentier@univ-perp.fr (M.-C.C.); cecile.antonelli@univ-perp.fr (C.B.-A.); 2 Université de Perpignan Via Domitia, LGDP-UMR5096, 58 avenue Paul Alduy, 66860 Perpignan, France

**Keywords:** degradome, 5′P, GMUCT, mRNA turnover, Arabidopsis, 5′-3′ degradation

## Abstract

The recent development of high-throughput technologies based on RNA sequencing has allowed a better description of the role of post-transcriptional regulation in gene expression. In particular, the development of degradome approaches based on the capture of 5′monophosphate decay intermediates allows the discovery of a new decay pathway called co-translational mRNA decay. Thanks to these approaches, ribosome dynamics could now be revealed by analysis of 5′P reads accumulation. However, library preparation could be difficult to set-up for non-specialists. Here, we present a fast and efficient 5′P degradome library preparation for Arabidopsis samples. Our protocol was designed without commercial kit and gel purification and can be easily done in one working day. We demonstrated the robustness and the reproducibility of our protocol. Finally, we present the bioinformatic reads-outs necessary to assess library quality control.

## 1. Introduction

Turnover of messenger RNAs (mRNAs) is a crucial and dynamic mean to control and alter gene expression to answer developmental and environmental cues in eukaryotes. Modulation of mRNA turnover rates, allows the change of their half-lives, hence permitting to modify their translation efficiency to answer cellular needs. In the cytosol, the so-called general mRNA turnover can decay transcripts through two pathways respectively degrading mRNAs from their 5’ or their 3’ extremity. The 5′-3′ decay process begins by the shortening of the polyA tail followed by the removal of the cap structure releasing molecules with 5′ monophosphate extremities (5′P). This step grants access to the body of the transcript to XRN exoribonucleases, XRN1 in yeast and XRN4 in *Arabidopsis thaliana* that nibble the mRNA [1].

The development of high-throughput technologies has allowed a better description of quantitative and qualitative changes of the polyadenylated transcriptome but has also permitted to get a deeper insight in mRNA lifecycle and regulatory mechanisms. In particular, the development of RNA degradome approaches extends our understanding of the role of mRNA turnover in proper gene expression. Different degradome approaches were developed but all of them are based on the capture of 5′ monophosphate decay intermediates. The initial approaches such as Degradome-seq [2], PARE (Parallel Analysis of RNA Ends [3]), or GMUCT (Genome-wide Mapping of Uncapped Transcripts [4]) are modified 5′RACE and were initially designed to identify endonucleolytic cleavages. However, recently, the discovery of a 5′-3′ co-translational decay of mRNAs in yeast through 5′P sequencing (5′P-seq) further emphasized the importance of mRNA decay in gene expression modulation and revived the interest in degradome data analyses [5].

**Figure 1:**
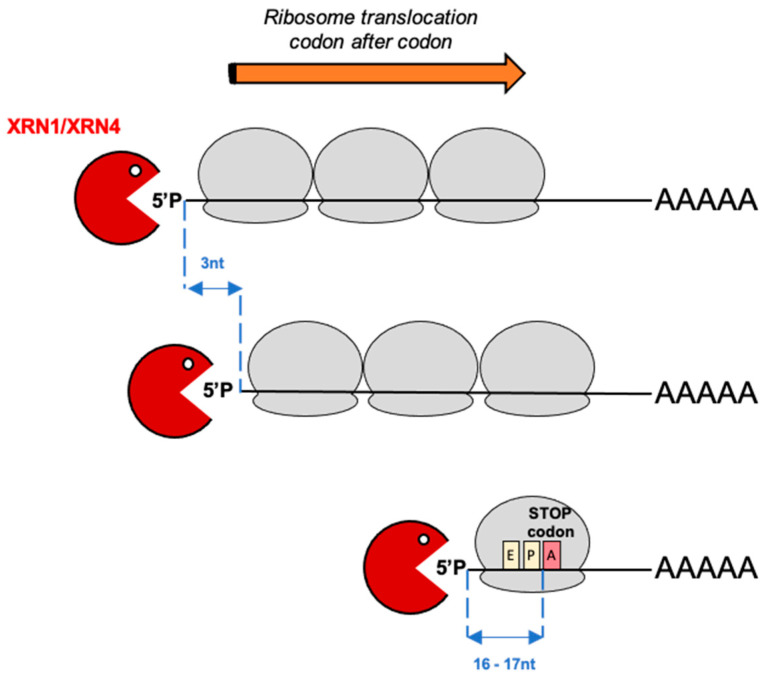
Representation of the co-translational mRNA decay pathway. The co-translational mRNA decay pathway was discovered in many organisms such as yeast, mammals or plants. For this process, decapping occurs on polysomes generating a 5′ monophosphate (5′P) extremity. The exoribonuclease XRN1/XRN4 immediately follows the last ribosome codon after codon and degrades the mRNA as it is being translated. A 3 nucleotide periodicity is thus observed between 5′P reads. As the termination step is slower than elongation, a general 5ʹP reads accumulation can be revealed 16–17 nucleotides upstream of stop codons. This distance corresponds exactly to a ribosome stalled at the A site.

The co-translational decay pathway was first found to exist in the yeast *Saccharomyces cerevisiae* through study of chosen model mRNAs [6]. The widespread effect and evolutionary conservation of this pathway was next revealed thanks to degradome approaches [5,7,8]. This process affects all transcripts and directly occurs on translating mRNAs that 5ʹ–3ʹ exoribonuclease chews chasing after the last translating ribosome (Figure 1). This follow-up leaves a 3-nucleotide distance between captured 5′P reads as the exoribonuclease movement is governed by the ribosome translocation codon after codon [5]. This pathway is well conserved and was discovered in many organisms such as yeast, mammals or plants using different degradome approaches [5,7,8]. Thus, in addition to endonucleolytic cleavages, degradome approaches also allow the capture of in vivo ribosome footprints and reveals classical translation marks such as a 3 nucleotide periodicity between 5′P reads and clear overaccumulation of reads 17 nucleotides upstream stop codon reflecting the last translating ribosome slowed down by the termination step [5]. In *Arabidopsis thaliana*, according to condition or tissue analyzed, these 5′P reads accumulation occurs at 16 or 17 nt before stop codons [7,8,9,10]. This distribution seems to be also dependant of the stop codon type [7].

Ribosome profiling (Ribo-Seq) was the current standard for investigation of ribosome dynamics. But now, many papers relate the use of degradome data to assess ribosome dynamics under various conditions at the level of translation initiation, elongation or termination. In yeast, 5′P-seq data reveal general translation termination pauses and novel codon-specific ribosomal pausings not detected by Ribo-Seq [5]. In addition, the role of eIF5A in translation termination and elongation was recently uncovered through degradome sequencing analyses [11]. The authors elegantly demonstrated that in the absence of eIF5A, ribosomes stall at proline stretches in addition to ribosome accumulation at stop codons. In the same manner, a functional connection between protein folding and translation elongation was recently revealed through the analysis of *ribosomal protein uL3* [W2555C] allele degradome data [12]. In this mutant, a clear ribosome accumulation around start codon could be detected. Recently, a user-friendly pipeline was released for degradome analysis allowing an interactive visualization of degradome data to facilitate data interpretation [10].

In plants, the co-translational decay pathway plays important roles in response to stress or across development. As an example, heat stress triggers a 5′ ribosome pausing inducing a massive and active 5′ to 3′ mRNA co-translational degradation in Arabidopsis at the basis of the reprogrammation of 1500 mRNA half-lives [13,14]. More recently, the degradome approach on polyA^+^ and polyA^−^ mRNAs uncovered the role of mRNA decay in nitrogen and dark stress responses [15]. In the same way, degradome approaches identified the repertoire of XRN4 co-translational decay targets and demonstrated the importance of co-translational decay across Arabidopsis seedling development [8]. By combining, degradome approach and polysome RNA sequencing, the authors also demonstrated that degradome data can be used to assess translation efficiency [8].

However, 5′P degradome library preparation could be time consuming and not trivial. Thus, we decided to improve library preparation to reduce time preparation and cost per sample. Here we propose a fast and efficient 5′P degradome library preparation thought the improvement of GMUCT approach [7]. We reduced the preparation time to 1 day from total RNA preparation to library quality control. Moreover, our protocol does not need any NGS library preparation kit reducing significantly library cost and allowing efficient library preparation from 50 μg to 0.5 μg of total RNA. In addition, we took advantage of this study to clearly present bioinformatic read-outs necessary to assess library quality prior to deeper bioinformatic analyses and to summarize the molecular events detectable by 5′P degradome data.

## 2. Results

### 2.1. Simplification of 5′P Degradome Library Preparation

**Figure 2:**
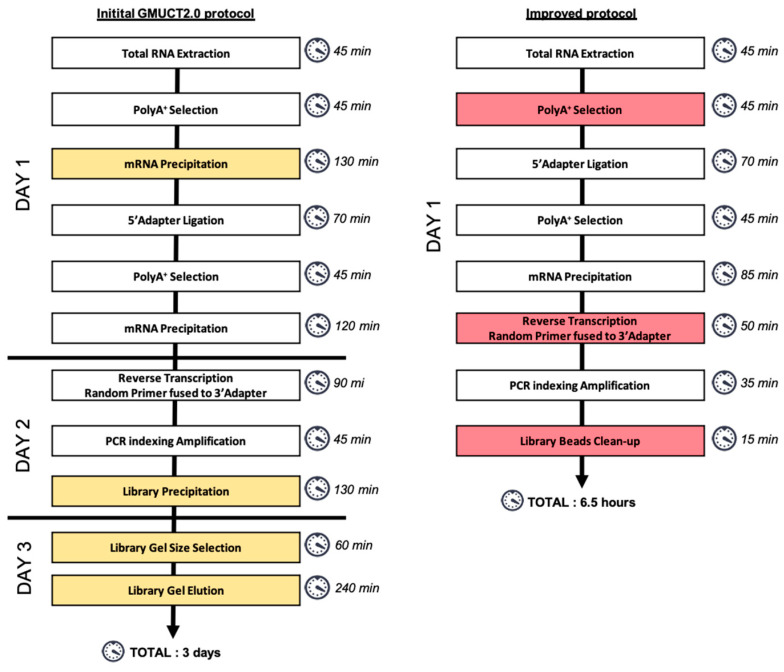
Comparison of the initial GMUCT2.0 and the improved protocol. The initial protocol takes 3 days and includes library gel size selection and many precipitations steps (highlighted in orange). To simplify GMUCT2.0 protocol, we adjusted volume reactions to limit precipitation steps, used a more efficient reverse transcriptase and replaced library gel size selection by a library beads clean-up (highlighted in red). Approximative duration time is indicated for each step.

We developed a fast and efficient 5′P degradome library preparation through the improvement of GMUCT2.0 approach [7]. The main workflow of 5′P degradome library prepation is presented in Figure 2. Briefly, after total RNA extraction, polyA^+^ mRNAs are purified prior to 5′adapter ligation (RA5). Excess of adapter is then removed by a second round of polyA^+^ selection. Reverse transcription is then performed using a random primer fused to a 3′adapter (RA3). Libraries are then amplified by PCR using specific primers anchored on RA5 and RA3. Finally, the library is cleaned-up. After library quality control and normalization, sequencing is performed in Single Read from the 5′extremity.

To simplify and reduce the cost per sample, we developed a protocol without NGS commercial kit contrary to GMUCT2.0. The main steps were mantained but we adjusted volume reactions to reduce precipitation times (Figure 2). For the first polyA^+^ purification step, we adjusted the final volume of elution, thus the 5′ ligation step can be directly done without prior mRNA precipitation. For reverse transcription, we used a more efficient and robust reverse transcriptase reducing the duration time. The most tricky part of initial GMUCT2.0 is the gel purification of the library. For non-experts, library gel purification could be difficult to set-up making reproducibility difficult. Thus, to improve this step, we developed a library purification based on clean-up magnetic beads allowing efficient and fast library preparation (Figure 2). This purification allows the removal of primers and adapter-adapter fragments. In this way, our protocol can be easily completed in 1 working day from total RNA extraction to library quality control with classical molecular biology reagents while initial GMUCT2.0 protocol takes 2–3 days (Figure 2). As our protocol does not include any gel size selection, no significant expertise in NGS library preparation is needed. Additionally, we estimated the cost is reduced by at least 3-fold compared to GMUCT 2.0 [7].

### 2.2. Validation of 5′P Degradome Library

**Figure 3:**
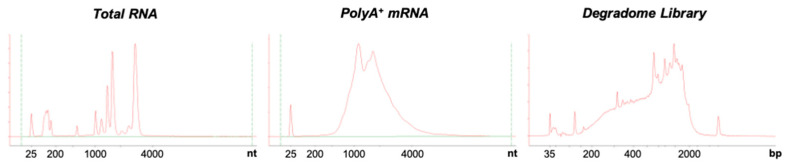
5′P degradome library quality control. Bionalyzer profiles of total RNA, mRNAs after PolyA+ purification and after library construction.

To validate our protocol, we firstly assessed total RNA, polyA^+^ mRNA and library quality control using bioanalyzer (Figure 3). Total and polyA^+^ mRNA samples present classical quality control profiles. Degradome library size ranges from 150 to 2000 pb as generally observed for this kind of library [4].

**Figure 4:**
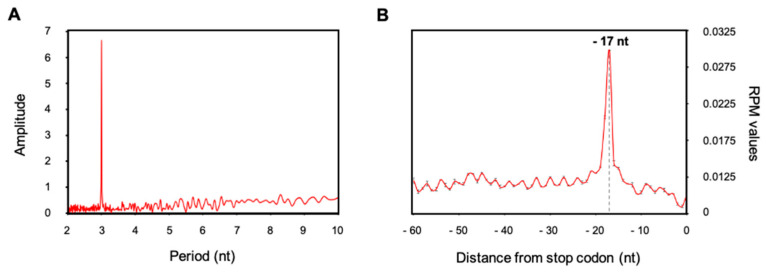
Read-outs of 5′P degradome library quality. Metagene analysis displaying periodicity between 5′ reads extremity (**A**) and 5′P reads accumulation around stop codon (**B**). N = 2, mean ± sd.

As our protocol gives a suitable degradome library profile, we constructed degradome libraries from two Arabidopsis biological replicates (Col0) 15-d-old seedlings. From the same biological replicates, we constructed libraries using our protocol and GMUCT 2.0 protocol [4]. The four libraries were prepared and sequenced together using a NextSeq 550 (SR 75). After sequencing, reads were trimmed to 50 nt and mapped to Arabidopsis TAIR10 genome. To assess the quality of our library preparation, two read-outs were followed: the periodicity between 5′P reads and their accumulation around stop codon (Figure 4). In fact, as degradome data can reveal co-translational decay, a 3-nt periodicity between 5′P reads and 5′P reads overaccumulation 17 nt before stop codon are expected as exoribonucleases follow the last translating ribosome codon after codon until the termination step. As expected, we found in our data a clear 3-nt periodicity between 5′P reads (Figure 4A) and a significant 5′P read accumulation 17 nt before stop codon (Figure 4B). These two read-outs assess the quality of library preparation and can be systematically used for new experiments.

**Figure 5:**
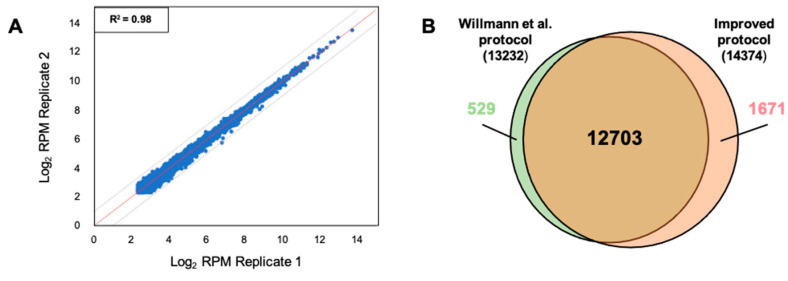
5′P degradome libraries are highly reproducible. (**A**) Correlation between read counts (rpm) obtained in replicate 1 versus replicate 2. (**B**) Transcripts identified in degradome library (RPM > 5) using Willmann et al. protocol (N = 13,232) and our protocol (N = 14,374).

Next, we assessed the reproducibility and the number of transcripts identified using our protocol as compared to the GMUCT 2.0 one. We determined the number of 5′P reads per transcript using HTSeq and retained only transcripts with an RPM (reads per milion) value higher than 5. We compared read counts between each replicate and observed a good correlation demonstrating the robustness of our protocol (Figure 5A). Our data were then compared with data produced by GMUCT 2.0 protocol [7]. For both protocols, more than 13,000 transcripts were identified (RPM > 5) (Figure 5B). Our protocol allows the identification of 96% of transcripts identified using GMUCT 2.0 protocol and the identification of 1671 additional transcripts. Taken together, these comparisons demonstrate the robustness and the reproducibility of our updated protocol.

**Figure 6:**
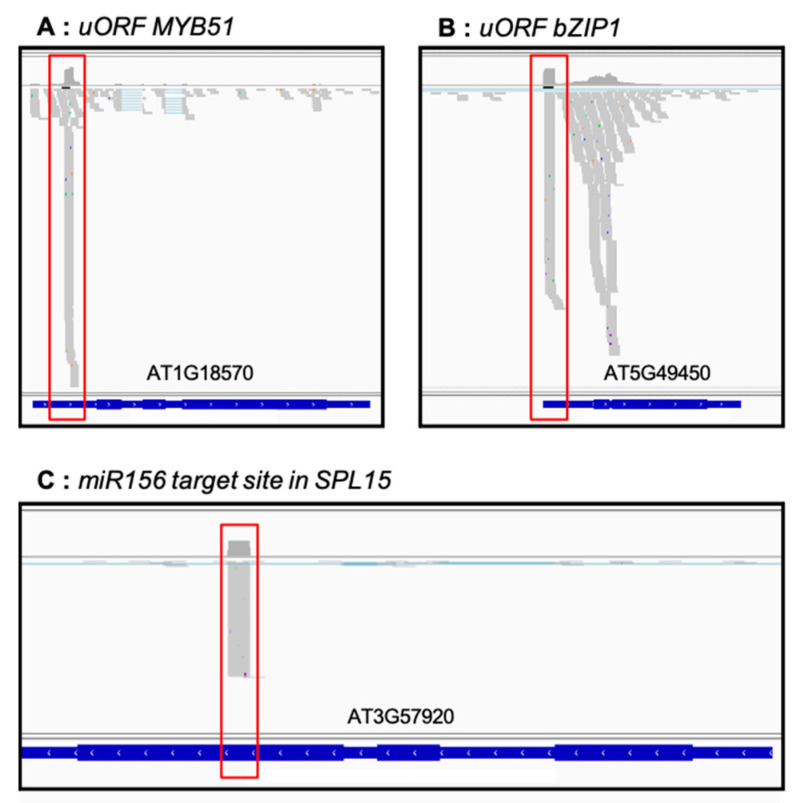
5′P degradome data allows identification of ribosome stacking at uORF or 5′P reads accumulation at miRNA cleavage site. Genome viewer of 5′P reads accumulation along At1g18570 (**A**), At5g49450 (**B**) and At3g57920 (**C**). Regions corresponding to uORF or miRNA cleavage site are highlighted in red. Grey marks correspond to 5′P reads accumulation.

As degradome data were proposed to reveal ribosome stalling at upstream open reading frame (uORF) or the identification of miRNA cleavages stites [4,9], we checked if these known events are detected in our dataset. Figure 6 presents 5′P reads accumulation along two transcripts known to contain uORF in their 5′UTR (At1g18570, At5g49450, Figure 6A,B) and 5′P reads accumulation at miRNA cleaveage site (miR156 target site in SPL15, Figure 6C).

As previously described, we succesfully detected 5′P reads accumulation in 5′UTR of At1g18570 and At5g49450 that corresponds to ribosome stalling at uORFs [9]. For miRNA cleavage site, we also observed 5′P reads accumulation on At3g57920 transcript exactly at the position of miR156 target site [7]. All together, these results demonstrate the robustness and the sensibility of our 5′P degradome protocol.

We also tested our protocol with different starting amounts of total RNA. We successfully obtained degradome libraries from 50 to 0.5 μg of total RNA. Table 1 presents the number of PCR cycles needed to obtain an efficiency library molarity according to amount of total RNA used.

## 3. Discussion

Here we present the development of an updated protocol for degradome library preparation. This protocol allows an easy preparation of library in one working day with common molecular biology reagents. To demonstrate robustness and reproducibility of our protocol, we analyzed 5′P reads accumulation around stop codons. We observed a clear accumulation of reads 17 nucleotides before stop codons. This accumulation corresponds to the last ribosome in termination step at A site and was classically observed in many degradome data and organisms [5,7,8,10]. Finally we demonstrated the reliability of our protocol by comparing degradome data produced by GMUCT2.0 protocol [4]. We found a significant overlap between protocols with the identification of more than 12,000 common transcripts. As it is now well accepted that 5′P degradome data can reveal ribosome dynamics, we propose that the 3nt periodicity and the 5′P reads accumulation around stop codons should be systematically tested to assess the quality of the library (Figure 4).

In addition to ribosome dynamics analysis, degradome data can reveal additional RNA pathways [9,16,17]. As an example, recent degradome data were used to reveal Exon Junction Complex (EJC) footprints in Arabidopsis, rice, worm and human [16]. Thanks to degradome data, the authors demonstrated the presence of in vivo EJC footprints allowing new research strategies for EJC-bound mRNAs. Degradome data reveal global ribosome stalling at termination step but additional ribosome stallings were also revealed. Hou and co-authors demonstrated that ribosome stalling can also occur on uORFs and CDS regions extending the use of degradome data [9].

Since the discovery of the co-translational decay pathway, degradome data are more and more used to reveal ribosomes dynamic in different conditions [5,8,11,12,18] and were already used in different organisms and plant species such as *Arabidopsis thaliana*, rice or soybean [9]. As this pathway is evolutionarily conserved and because only total RNA is necessary to prepare degradome library, we think that degradome data can be easily developed for non-model species to expand our knowledge on translation and mRNA decay regulation.

## 4. Materials and Methods

**Plant culture**

Arabidopsis (*Arabidopsis thaliana*) Col-0 seedlings were grown during 15 days on synthetic Murashige and Skoog medium (Duchefa) containing 1% (*w/v*) sucrose and 0.8% (*w/v*) plant agar at 22 °C under a 16-h-light/8-h-dark regime.

**Oligonucleotides**

***RA5*** *(HPLC purified, 12 mM, order for synthesis)*

5′-rGrUrUrCrArGrArGrUrUrCrUrArCrArGrUrCrCrGrArCrGrArUrC-3′

***RA3*** *(HPLC purified, 20 μM, order for synthesis)*

5′-CTGGAGTTCAGACGTGTGCTCTTCCGATCTNNNNNN-3′

***PCR Forward Primer*** *(HPLC purified, 10 μM, order for synthesis)*

5′-AATGATACGGCGACCACCGAGATCTACACGTTCAGAGTTCTACAGTCCG*A-3′

blue: P5 sequence, red: 5′adapter sequence, *phosphorothioate bond

***PCR Reverse Index Primer*** *(NEBNext Multiplex Oligos, E7335S or E7500S or E7710S or E7730S for multiplexing up to 48 samples)*

5′-CAAGCAGAAGACGGCATACGAGAT**XXXXXX**CTGGAGTTCAGACGTGTGCTCTTCCGATC*T-3′

orange: P7 sequence, green: 3′adapter sequence, **XXXXXX**: index sequence, *phosphorothioate bond

**Total RNA extraction**

*Expected Yield: around 50 μg for 100 mg of seedling tissue, Estimated time: 45 min.*

Total RNA was extracted using Monarch Total RNA Miniprep Kit (New England Biolabs) according to manufacturer’s instructions including DNAse treatment. 100 mg of tissue powder were used per column. After extraction, total RNA was quantified using Qubit HS RNA Kit (Fisher Scientific) and the quality was assessed using Bioanalyzer RNA Nano Kit (Agilent).

**PolyA^+^ purification**

*Expected Yield: 1%, Estimated time: 45 min*

mRNAs were purified using Dynabeads mRNA Direct Kit (Fisher Scientific) according to manufacturer’s instructions. 50 μg of total RNA were purified with 150 μL of beads. Final elution wad performed with 30 μL of Elution Buffer. After purification, mRNA was quantified using Qubit HS RNA Kit (Fisher Scientific) and quality was assessed using Bioanalyzer RNA Nano Kit (Agilent).

**5′Adapter ligation**

*Estimated time: 70 min + 45 min + 85 min*

For 5′Adapter (RA5) ligation, 400 ng of mRNA was combined on ice with 12 pmol of RNA 5′Adapter (RA5) in a 200 µL PCR tube in a final volume of 26 μL. Tube was incubated 2 min at 70 °C and immediately placed on ice for at least 2 min. Adapter ligation was performed using T4 RNA Ligase 1 (New England Biolabs). Then, 24 μL of master mix containing 5 μL of 10X T4 RNA Ligase Reaction, 12 μL of 50% PEG8000, 1 μL of T4 RNA Ligase 1 (10 units), 1 μL of RNase Inhibitor (40 units) and 5 μL of 10 mM ATP were added to each sample. The mixture was then incubated 1 h at 25 °C in a preheated thermal cycler. After incubation, an additional mRNA purification was performed to remove RA5 adapter excess. Ligated mRNAs were then precipitated by adding 10 μL of 3M Ammonium Acetate pH 5.4, 25 μg of glycogen, and 300 μL of EtOH 100%. After incubation for 30 min at −80 °C, the tube was centrifugated at 16,000× *g* for 45 min at 4 °C. Pellet was washed with 750 μL of cold 80% EtOH. Finally, pellet was resuspended in 7.5 μL of RNAse/DNAse free water (Fisher Scientific).

**Reverse-transcription using RA3 primer**

*Estimated time: 50 min*

Reverse transcription was performed using Superscript IV RT kit (Fisher Scientific) according to manufacturer’s instructions using 7.5 μL of ligated mRNA and 1.3 μL of RA3 primer (20 μM).

**PCR Amplification**

*Estimated time: 35 min*

PCR was performed by combining 20 μL of cDNA, 2.5 μL of PCR Reverse Index Primer (10 μM), 2.5 μL of PCR Forward Primer (10 μM), 50 μL of LongAmp Taq 2X Master Mix (New England Biolabs) and 25 μL of H_2_0. Amplification was performed with 11 cycles of denaturation at 94 °C for 15 s, annealing at 60 °C for 30 s and extension at 70 °C for 60 s followed by 5 min of extension at 70 °C.

**Library Purification**

*Estimated time: 15 min*

Library was purified with SPRISelect beads (Beckman Coulter) according to manufacturer’s instructions with a ratio of 0.9X (90 μL). Library is eluted in a final volume of 20 μL. Library concentration was assessed using Qubit dsDNA HS kit (Fisher Scientific) and the quality was assessed using Bioanalyzer DNA High Sensitivity Kit (Agilent).

**Sequencing**

Libraries were normalized, pooled and sequenced in SR75 on a NextSeq550 according to Illumina’s instructions.

**Bioinformatic analysis**

Raw reads were trimmed to 50 pb using Trimmomatic v0.36 [19]. Trimmed reads were filtered out from reads corresponding to chloroplastic, mitochondrial, ribosomal and small RNA sequences using bowtie2 v2.3.4 [20] in ‘sensitive-local’ mode. Reads mapping against TAIR10 genome and corresponding gtf file annotations were performed using Hisat2 v2.0.3 [21]. Only unique mapped reads were kept using samtools v1.9 with option ‘-q 10’ [22]. Read count was performed using htseq-count v0.11.2 [23] in ‘union’ mode and normalized by total of mapped reads (reads per millions, RPM). For 5′P reads metagene analysis, alignment files (bam format) were converted into bed files containing only the first nucleotide of each read using bedtools2 v2.28 [24]. Read count around the stop region per bin of 1pb was performed using bedcoverage and normalized by total of mapped reads in the defined window. For 3 nucleotide periodicity analysis, raw data were analyzed using 5′P seq software [10]. Data corresponding to 3 nucleotide periodicity were retrieved from “fft_signal” files.

## Figures and Tables

**Table 1:** Recommended amplification according to the amount of total RNA used as a starting point. After amplification, a library molarity between 10 and 15 nM is expected. This molarity is adequate for following sequencing steps.

Starting Material (Total RNA)	Recommended PCR Cycles	Yield ng/μl	Library Molarity (nM)
50 μg	10–11	3–4	10–15
5 μg	15–16	3–4	10–15
0.5 μg	16–17	3–4	10–15

## Data Availability

The accession number for the 5′Pdegradome data reported in this article is NCBI Bioproject PRJNA671817.
